# Supplementary material for: What is the ‘voltage drop’ when an effective health promoting intervention for older adults—Choose to Move (Phase 3)—Is implemented at broad scale?
Source: PLoS One. 2023 May 5;18(5):e0268164. doi: 10.1371/journal.pone.0268164 (PMC10162521; doi:10.1371/journal.pone.0268164)
Supplement: S1 Table — Baseline socio-demographic characteristics of participants who completed the Choose to Move intervention, those lost to follow-up and those in Cycle 8 (Winter 2020 cohort, excluded from the primary analysis). Values are n (%) or mean (standard deviation, SD). (DOCX) [file pone.0268164.s001.docx]

**S1 Table. Comparison of descriptive characteristics between participants who completed Choose to Move, those lost to follow-up and those in Cycle 8.** Baseline socio-demographic characteristics of participants who completed the CTM intervention, those lost to follow-up and those in Cycle 8 (excluded from the primary analysis). Values are n (%) or mean (standard deviation, SD).

|  | **Completed CTM & evaluation (6 months)** | **Lost to follow-up**  **(at 6 months)** | **Cycle 8 participants** | **p-value^a^** |
| --- | --- | --- | --- | --- |
| Participants, n (men / women) | 705 (142 / 563) | 308 (54 / 254) | 203 (41/162) | 0.604 |
| % (men) | 20.1% | 17.5% | 20.2% |  |
| Age (years) | 73.0 (6.2) | 72.7 (6.6) | 72.3 (6.1) | 0.369 |
| Age category |  |  |  |  |
| 54-74 years | 454 (64.4%) | 196 (63.6%) | 129 (63.9%) | 0.970 |
| >75 years | 251 (35.6%) | 112 (36.4%) | 73 (36.1%) |  |
| Delivery partner, n (BCRPA / YMCA) | 430 / 275 | 201 / 107 | 137 / 66 | 0.162 |
| BMI, kg/m^2^ | 29.4 (6.3) | 29.3 (6.5) | 29.5 (6.3) | 0.936 |
| Ethnicity, n (%) |  |  |  |  |
| White | 636 (90.2%) | 257 (83.4%) | 176 (86.7%) | 0.034 |
| Asian | 39 (5.5%) | 27 (8.8%) | 17 (8.4%) |  |
| Other | 30 (4.3%) | 24 (7.8%) | 10 (4.9%) |  |
| Educational attainment, n (%) |  |  |  |  |
| Secondary or less | 190 (27.0%) | 92 (30.0%) | 37 (18.2%) | 0.023 |
| Some trade, technical school or college | 245 (34.9%) | 111 (36.2%) | 89 (43.8%) |  |
| Some university | 268 (38.1%) | 104 (33.9%) | 77 (37.9%) |  |
| Chronic Conditions, n (%) |  |  |  |  |
| 0 | 116 (16.5%) | 34 (11.0%) | 27 (13.4%) | 0.062 |
| 1 | 249 (35.3%) | 123 (39.9%) | 63 (35.8%) |  |
| ≥ 2 | 340 (48.2%) | 151 (49.0%) | 112 (49.6%) |  |
| Mental Health Conditions, n (%) |  |  |  |  |
| 0 | 467 (76.4%) | 186 (72.1%) | 151 (74.8%) | 0.765 |
| 1 | 90 (14.7%) | 45 (17.4%) | 32 (15.8%) |  |
| ≥ 2 | 54 (8.8%) | 27 (10.5%) | 19 (9.4%) |  |
| Mobility limitations (walk and/or stair), n (%) |  |  |  |  |
| Yes | 317 (45.1%) | 142 (46.6%) | 79 (39.1%) | 0.223 |
| No | 386 (54.9%) | 163 (53.4%) | 123 (60.9%) |  |
| Self-rated health, n (%) |  |  |  |  |
| Very poor, poor or fair for age | 311 (44.2%) | 150 (49.2%) | 106 (53.0%) | 0.060 |
| Good or excellent for age | 392 (55.8%) | 155 (50.8%) | 94 (47.0%) |  |
| Physical activity | 2.4 (2.1) | 2.4 (2.0) | 2.1 (1.9) | 0.120 |
| Social isolation score | 11.0 (3.0) | 11.0 (3.2) | 10.8 (3.3) | 0.841 |
| Loneliness | 4.4 (1.6) | 4.6 (1.9) | 4.5 (1.7) | 0.186 |
| Health Status (EQ-5D-5L) | 0.794 (0.138) | 0.779 (0.150) | 0.788 (0.159) | 0.324 |
| VAS | 70.4 (17.9)^b^ | 67.7 (19.5) | 67.3 (16.3) | 0.029 |

BCRPA: British Columbia Parks and Recreation Association; YMCA: Young Men’s Christian Association; VAS: Visual Analog Scale

^a^ *p* values calculated using two-tailed chi-squared or Fisher’s exact test for categorical variables (sex, age category, ethnicity, education, chronic conditions, mobility limitations) and analysis of variance for continuous variables (body mass index and impact variables)

^b^ significantly different from the Lost to Follow-up (p=0.033) and Cycle 8 (p=0.037) groups
