# Supplementary material for: What is the ‘voltage drop’ when an effective health promoting intervention for older adults—Choose to Move (Phase 3)—Is implemented at broad scale?
Source: PLoS One. 2023 May 5;18(5):e0268164. doi: 10.1371/journal.pone.0268164 (PMC10162521; doi:10.1371/journal.pone.0268164)
Supplement: S2 Table — Adjusted means (95% confidence interval) for impact outcome measures by time point and age group in Choose to Move Phase 3 –Cycle 8 (Winter 2020 cohort; excluded from primary analysis due to impact of COVID-19 on program delivery). (DOCX) [file pone.0268164.s002.docx]

**S2 Table. Results of linear mixed model for impact outcome measures for Choose to Move participants in Cycle 8 (Winter 2020 cohort).** Adjusted means (95% confidence interval) for impact outcome measures by time point and age group in the Choose to Move Phase 3 – Winter 2020 cohort (excluded from primary analysis due to impact of COVID-19 on program delivery).

|  | **Months** | **Winter 2020 cohort sample size:**  **Full cohort / <75 years / > 75 years** | **Full Winter 2020 cohort** | **Winter 2020 <75 years** | **Winter 2020 ≥ 75 years** | **p-value^a^**  **Full sample**  **0-3 mos**  **0-6 mos** | **p-value^a^**  **<75 yrs**  **0-3 mos**  **0-6 mos** | **p-value^a^**  **≥75 yrs**  **0-3 mos**  **0-6 mos** |
| --- | --- | --- | --- | --- | --- | --- | --- | --- |
| **Physical activity (# d/wk>30 min)** | 0 | 199 / 128 / 70 | 2.1 (1.9, 2.4) | 2.0 (1.7, 2.3) | 2.4 (2.0, 2.8) |  |  |  |
|  | 3 | 177 / 113 / 64 | 2.6 (2.0, 3.2) | 2.7 (2.0, 3.4) | 2.5 (1.5, 3.5) | 0.329 | 0.200 | >0.99 |
|  | 6 | 147 / 91 / 56 | 2.9 (2.2, 3.5) | 2.9 (2.0, 3.7) | 2.8 (1.8, 3.7) | 0.098 | 0.128 | >0.99 |
| **Mobility [n, (%) reporting any limitation]** | 0 | 202 / 129 / 72 | 79 (39.1) | 45 (34.9) | 34 (47.2) |  |  |  |
|  | 3 | 177 / 113 / 64 | 64 (36.2) | 36 (31.9) | 28 (43.8) | 0.554 | 0.619 | 0.685 |
|  | 6 | 148 / 91 / 57 | 51 (34.5) | 27 (29.7) | 24 (42.1) | 0.374 | 0.417 | 0.562 |
| **Social Isolation**  **(score, 0–15)** | 0 | 202 / 129 / 72 | 10.8 (10.4, 11.3) | 10.6 (10.0, 11.1) | 11.3 (10.6, 12.1) |  |  |  |
|  | 3 | 177 / 113 / 64 | 8.8 (8.3, 9.3) | 8.8 (8.2, 9.4) | 8.8 (8.1, 9.6) | <0.001 | <0.001 | <0.001 |
|  | 6 | 148 / 91 / 57 | 9.4 (8.9, 9.9) | 9.2 (8.6, 9.8) | 9.6 (8.8, 10.5) | <0.001 | <0.001 | 0.001 |
| **Loneliness**  **(score, 3–9)** | 0 | 200 / 128 / 71 | 4.5 (4.3, 4.7) | 4.7 (4.3, 5.0) | 4.2 (3.8, 4.6) |  |  |  |
|  | 3 | 168 / 111 / 57 | 4.8 (4.5, 5.1) | 5.0 (4.7, 5.3) | 4.5 (4.0, 4.9) | 0.042 | 0.086 | 0.719 |
|  | 6 | 140 / 90 / 50 | 4.7 (4.4, 4.9) | 4.8 (4.4, 5.1) | 4.5 (4.0, 4.9) | 0.615 | >0.99 | 0.721 |
| **Health status**  **(EQ-5D-5L)** | 0 | 201 / 129 / 71 | 0.789 (0.771, 0.807) | 0.789 (0.760, 0.806) | 0.800 (0.769, 0.831) |  |  |  |
|  | 3 | 176 / 113 / 63 | 0.803 (0.761, 0.845) | 0.800 (0.748, 0.851) | 0.809 (0.735, 0.883) | >0.99 | >0.99 | >0.99 |
|  | 6 | 146 / 91 / 55 | 0.797 (0.750, 0.844) | 0.785 (0.725, 0.846) | 0.819 (0.745, 0.893) | >0.99 | >0.99 | >0.99 |
| **VAS**  **(EQ-5D-5L)** | 0 | 201 / 129 / 72 | 67.2 (65.1, 69.3) | 66.0 (63.3, 68.6) | 69.4 (65.7, 73.0) |  |  |  |
|  | 3 | 177 / 113 / 64 | 70.6 (65.7, 75.4) | 69.3 (63.3, 75.2) | 72.9 (64.7, 81.2) | 0.505 | 0.838 | >0.99 |
|  | 6 | 148 / 91 / 57 | 69.8 (64.3, 75.2) | 70.2 (63.1, 77.3) | 69.2 (60.5, 77.8) | >0.99 | 0.715 | >0.99 |

d/wk: days/week; Social isolation: higher score indicates a larger social network; Loneliness: lower score indicates lower feelings of loneliness; EQ-5D-5L Health Status and Visual Analog Scale (VAS): higher score indicates better health status

^a^ *p* values for continuous variables were calculated using a ﬁtted linear mixed effects models for each continuous impact variable with time (0, 3 and 6 months) as a categorical predictor. Fixed effects were sex and age category (60-74 years, > 75 years); additional covariates were delivery partner, baseline mobility limitation (yes/no), number of chronic conditions (0, 1, ≥2), education and body mass index. Adjusted values were calculated at each time point using the margins command in Stata with a Bonferroni adjustment to account for multiple comparisons between and within age groups. *p* values for mobility limitations were calculated using chi-squared tests with Bonferroni adjustment to account for multiple comparisons between time points (signiﬁcance at: 0.05/3 = 0.017).
